# A Genetic Analysis of the Functional Interactions within *Mycobacterium tuberculosis* Single-Stranded DNA Binding Protein

**DOI:** 10.1371/journal.pone.0094669

**Published:** 2014-04-10

**Authors:** Kervin Rex, Sanjay Kumar Bharti, Shivjee Sah, Umesh Varshney

**Affiliations:** 1 Department of Microbiology and Cell Biology, Indian Institute of Science, Bangalore, India; 2 Jawaharlal Nehru Centre for Advanced Scientific Research, Bangalore, India; Saint Louis University, United States of America

## Abstract

Single-stranded DNA binding proteins (SSBs) are vital in all organisms. SSBs of *Escherichia coli* (*Eco*SSB) and *Mycobacterium tuberculosis* (*Mtu*SSB) are homotetrameric. The N-terminal domains (NTD) of these SSBs (responsible for their tetramerization and DNA binding) are structurally well defined. However, their C-terminal domains (CTD) possess undefined structures. *Eco*SSB NTD consists of β1-β1′-β2-β3-α-β4-β45_1_-β45_2_-β5 secondary structure elements. *Mtu*SSB NTD includes an additional β-strand (β6) forming a novel hook-like structure. Recently, we observed that *Mtu*SSB complemented an *E. coli Δssb* strain. However, a chimeric SSB (mβ4-β5), wherein only the terminal part of NTD (β4-β5 region possessing L_45_ loop) of *Eco*SSB was substituted with that from *Mtu*SSB, failed to function in *E. coli* in spite of its normal DNA binding and oligomerization properties. Here, we designed new chimeras by transplanting selected regions of *Mtu*SSB into *Eco*SSB to understand the functional significance of the various secondary structure elements within SSB. All chimeric SSBs formed homotetramers and showed normal DNA binding. The mβ4-β6 construct obtained by substitution of the region downstream of β5 in mβ4-β5 SSB with the corresponding region (β6) of *Mtu*SSB complemented the *E. coli* strain indicating a functional interaction between the L_45_ loop and the β6 strand of *Mtu*SSB.

## Introduction

Single-stranded DNA binding protein (SSB) binds single-stranded DNA in a sequence independent manner during major DNA transactions such as DNA replication, repair and recombination [1]–[5]. Besides their crucial function in DNA transactions, they protect transiently generated single-stranded DNA (ssDNA) from nucleases or chemical attacks [6]. The eubacterial SSBs contain subunits with a similar basic fold, but may exhibit variations in their quaternary association [7]. SSBs possess an oligonucleotide-binding fold (OB-fold) in the N-terminal domain responsible for their oligomerization and DNA binding. The conserved C-terminal acidic tail of SSBs is important in protein-protein interactions [8]–[11]. One of the features of *Eco*SSB, important for its *in vivo* function, is the dynamic transition in its modes of DNA binding [6], [12]. SSB binds to ∼35 nucleotides by two of its subunits known as SSB_35_ mode and is required for unlimited cooperatively. While all the four subunits bind to ∼56 or ∼65 nucleotides in a limited cooperative manner known as SSB_56_ or SSB_65_ modes, respectively [13]–[16].

**Figure 1:**
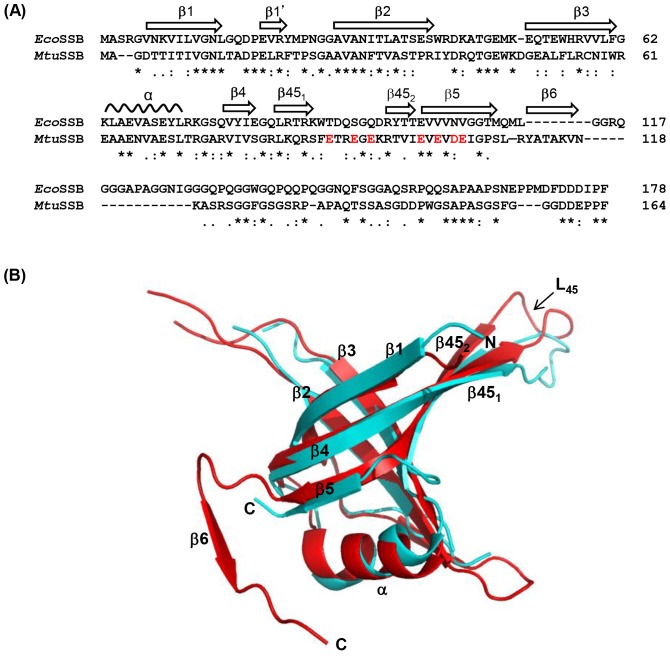
Comparison of *Eco*SSB and *Mtu*SSB. (A) *Eco*SSB and *Mtu*SSB sequences were aligned with ClustalW program. Identical amino acid residues (*), very similar amino acid residues (:) and similar amino acid residues (.) are indicated. Secondary structural elements (α helix and β strands) are shown as per *Eco*SSB nomenclature [17]. Acidic amino acids in *Mtu*SSB L_45_ region are highlighted in ‘red’. (B) DNA binding domains of *Eco*SSB and *Mtu*SSB tertiary structures were superposed using Pymole. Various secondary structural elements mentioned in Fig. 1A are indicated. L_45_ loop in both the SSBs (connecting β45_1_ and β45_2_) are also indicated.

The crystal structures of SSB in free and DNA bound forms have provided valuable information to understand their function [17], [18]. *Eco*SSB monomer consists of an N-terminal domain (∼115 amino acids) of defined structure, and the C-terminal domain whose three dimension structure is not available. The tertiary structure of the N-terminal domain of *Eco*SSB is defined by the presence of β1-β1′-β2-β3-α-β4-β45_1_-β45_2_-β5 secondary structure elements (Fig. 1). In the X-ray crystal structure, one of the β hairpin loops (L_45_) with well-defined electron density connects β4 and β5. Structural studies of *Eco*SSB suggested that its quaternary association is mediated by the L_45_ loops as well as by the six-stranded β-sheets formed by the dimers [17]. Furthermore, the L_45_ loop undergoes a significant change upon binding to DNA [18]. Functional importance of this movement, however, remains unclear.

*Mtu*SSB shares ∼30% identity and ∼39% similarity with *Eco*SSB in its primary sequence. The secondary structure involved in OB-fold is very similar in the two SSBs except for the presence of a novel β6 strand (numbered according to *Eco*SSB, 17) downstream of the β5 in *Mtu*SSB (Fig. 1). While both the SSBs share overlapping tertiary structures, there are notable variation in their quaternary associations due to the presence of the β6 strand in *Mtu*SSB [19]. Although a role for β6 strand in providing stability through the formation of a clamp like structure has been suggested in the mycobacterial SSBs [19]–[21] its biological importance is unknown.

Recently, using an *in vivo* assay wherein replication of the resident *ssb* support plasmid in an *E. coli* strain deleted for its chromosomal copy of *ssb* gene could be selectively blocked, we showed that overexpression of *Mtu*SSB complemented *E. coli* [22]. However, a chimeric SSB (mβ4-β5), wherein the β4-β5 region (which possess the L_45_ loop) of *Eco*SSB was replaced with the corresponding secondary structure elements of *Mtu*SSB, did not complement the strain [22]. This suggested that the L_45_ loop might be involved in specific interactions within *Mtu*SSB. In this study, we have designed additional chimeric constructs to uncover the importance of such interactions between the *Mtu*SSB L_45_ loop and the novel β6 strand for its function in *E. coli*.

## Material and Methods

### DNA oligomers, bacterial strains and media

DNA oligomers (Table 1) were obtained from Sigma-Aldrich, India. *E. coli* strains (Table 1) were grown in Luria-Bertani (LB) medium. LB-agar contained 1.6% (w/v) agar (Difco, USA) in LB. Ampicillin (Amp, 100 μg ml^−1^), kanamycin (Kan, 25 μg ml^−1^), tetracycline (Tet, 7.5 μg ml^−1^), or chloramphenicol (Cam, 15 μg ml^−1^) were added to growth media as required.

**Table 1:** List of strains, plasmids and DNA oligomers.

Strain/plasmids/DNA oligomer	Details	References
***E. coli*** ** strains**		
RDP 317-1 (or RDP 317)	*E. coli* (Δ*ssb*::*kan*) harboring pHYD*Eco*SSB (ColE1 *ori*, Cam^R^) whose replication is dependent upon the presence of IPTG.	[22]
TG1	An *E. coli* K strain, *supE hsd*Δ5 *thi* Δ(*lac-proAB*) F' [*traD*36 *proAB^+^ lacI^q^ lacZ*ΔM15]	[23]
BL21 (DE3)	Harbors T7 RNA polymerase gene under the control of LacI	Novagen
**Plasmids**		
pTrc*Eco*SSB	pTrc99C containing *Eco-ssb* ORF	[26]
pTrc*Eco*SSB (G114A)	pTrc99C containing *Eco-ssb* ORF wherein G114A mutation was incorporated to generate NheI site.	[22]
pTrc*Mtu*SSB	pTrc99C containing *Mtu-ssb* ORF	[26]
pTrc*Mtu*SSB(R111A)	pTrc99C containing *Mtu-ssb* ORF wherein R111A mutation was generated to create NheI site.	[22]
pHYD*Eco*SSB	Derived from pHYD1621 containing IPTG dependent ColE1 *ori* of replication. EcoRV to PstI fragment from pTrc*Eco*SSB was cloned into Ecl136II and PstI digested pHYD1621.	[22]
pBAD/HisB	pBAD/HisB plasmid (ColE1 *ori*, Amp^R^). An expression vector containing arabinose inducible promoter.	Invitrogen
pBADmβ4-β5(acidic)	pBAD containing chimeric mβ4-β5 SSB [22], wherein E_90_, T_91_, E_95_, K_96_, E_103_, D_105_, and E_106_ of *Mtu*SSB was replaced with T_90_ D_91_, Q_95,_ D_96_, V_103_ and N_105_ V_106_, respectively with *Eco*SSB specific sequences.	This work
pBADmβ4-β6	pBAD containing chimeric SSB wherein the first 73 amino acids are from *Eco*SSB (containing R73A mutation), amino acids from 74 to 131 from *Mtu*SSB and remaining 132 to 179 *Eco*SSB.	This work
pBADmβ1-α	pBAD containing chimeric SSB wherein the first 73 amino acids are from *Mtu*SSB, and the remaining amino acids (74 to 177) are from *Eco*SSB.	This work
pBADmβ6	pBAD containing chimeric SSB wherein the first 113 amino acids are from *Eco*SSB, amino acids from 114 to 133 are from *Mtu*SSB(R114A) and remaining 134 to 181 amino acids are from *Eco*SSB.	This work
pBADmβ6-CTD	pBAD containing mβ6 SSB wherein amino acids, 114 to 167 are from *Mtu*SSB(R114A).	This work
pBADmCTD	pBAD containing chimeric SSB wherein the first 128 amino acids are from *Eco*SSB, and remaining amino acids (129 to 164) are from *Mtu*SSB.	This work
**DNA oligomer (5′-3′)**		
*Eco*SSB-NheI-Fp	catgcagatgctagctggtcgtcaggg	[22]
*Eco*SSB-NheI-Rp	ccctgacgaccagctagcatctgcatg	[22]
*Eco*SSB-Fp	ggaattcaccatggccagcagagg	[22]
*Eco*SSB-XmaI-Fp	agcgaatatctggcccggggttctcaggtt	This work
*Mtu*SSB-NheI-FP	ttgggccttcgctagcgtacgccaccgc	[22]
*Mtu*SSB-NheI-Rp	gcggtggcgtacgctagcgaaggcccaa	[22]
pTrc-Bcl-Rp	ggctgttttggcggatgagaga	[22]
pTrc-Fp	taacaagcttacacaggaaacag	[22]
mβ4-β5 (acidic)-Fp	gtcgtttacagaccgttcgggccaggaccgcaccgtcatcgaggtcgtggtcaatgtgattggg	This work
mβ4-β5 (acidic)-Rp	cccaatcacattgaccacgacctcgatgacggtgcggtcctggcccgaacggtctgtaaacgac	This work
79 mer ssDNA	gcactagtgcggatagccccgtgttgttgtctgacccccgaccccgacggcaatgcggggcaatcccctggaggcctgc	[22]

### Cloning, overexpression, purification and gel filtration analysis of SSBs

Standard recombinant DNA methods and site directed mutagenesis [23] were used to generate chimeric SSBs (Table 1, and Methods S1). SSB open reading frames were subcloned into pTrc99C, pBAD/HisB and pET11d vectors, purified and stored in 50 mM Tris-HCl, pH 8.0, 0.1 mM Na_2_EDTA, 500 mM NaCl and 10% glycerol [22]. Oligomeric status of SSBs was determined by gel filtration chromatography [22], [24].

### Electrophoretic mobility shift assays (EMSA)

SSB tetramers (0.2, 2 and 10 pmol) were mixed with 5′ [^32^P] - end labeled 79mer DNA oligomer (1 pmol, ∼20,000 cpm) in 15 μl reactions containing 20 mM Tris-HCl, pH 8.0, 50 mM NaCl, 5% glycerol (v/v) and 50 μg/ml BSA, incubated for 30 min at 4°C and electrophoresed on 8% native-PAGE (30∶0.5, acrylamide:bisacrylamide) using 1× TBE (Tris-Borate-Na_2_EDTA) for 1–2 h at 15 V cm^−1^ in cold room, and visualized by BioImage Analyzer (FLA5000, Fuji).

### Complementation analysis

The complementation assays were performed using a recently described revised plasmid bumping method [22]. Briefly, the pBAD based expression constructs were introduced into *E. coli* RDP317-1 harboring pHYD*Eco*SSB as support plasmid (ColE1 *ori*, Cam^R^, whose replication is dependent on the presence of isopropyl-β-D-thiogalactopyranoside, IPTG) and the transformants were selected on LB agar containing Kan, Amp and 0.02% arabinose (or Kan, Amp and 0.5 mM IPTG, as control). The isolated colonies were streaked on LB agar containing Kan and Amp with various concentration of arabinose.

### Growth curve analysis

Freshly isolated transformants were inoculated in LB containing Kan, Amp and 0.02% arabinose to obtain late stationary phase cultures; and inoculated at 0.1% level in LB containing Kan, Amp and arabinose (as indicated) in the honeycomb plates. The growth was recorded at 600 nm using Bioscreen C growth reader (OY growth, Finland) at 37°C on an hourly basis. Average values (±SEM) were plotted.

### Microscopic studies

Freshly isolated transformants of *E. coli Δssb* strain harboring pBAD based SSB constructs were grown to log phase (7–9 h in 2 ml LB containing arabinose). Bacterial cells were collected by centrifugation, fixed with 4% paraformaldehyde, kept on poly-L-lysine treated multi-well slide, washed with PBS and visualized in fluorescence microscope (ZEISS, Axio Imager) with a 100× objective lens [22].

## Results

### Experimental rationale and generation of SSB chimeras

The N-terminal domain of *Eco*SSB is defined by β1-β1′-β2-β3-α-β4-β45_1_-β45_2_-β5 as its secondary structure elements (Fig. 1A). The N-terminal domain of *Mtu*SSB, in addition possesses a β6 strand (Fig. 1), which causes a notable variation in its quaternary structure by the formation of a clamp like structure at the dimeric interface of the interacting subunits [19]. The C-terminal domains of both the SSBs possess acidic tails important in protein-protein interactions during various DNA transactions [8]–[11].

**Figure 2:**
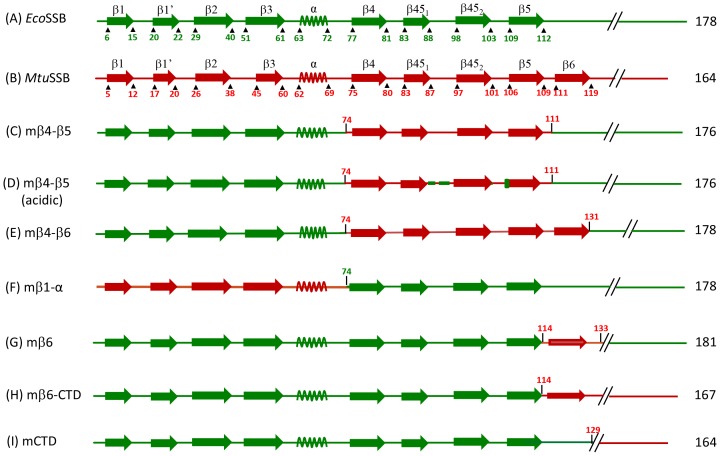
Schematic representation of various SSB constructs. Secondary structure elements of N-terminal domain of *Mtu*SSB and *Eco*SSB are represented in red and green, respectively. The beginning and end of structural unit are also numbered in the same color. The C-terminal domains are shown by discontinuous lines. SSB chimeras are shown in respective colors.

Recently, we observed that *Mtu*SSB sustained *E. coli* for its essential function of SSB [22]. However, the mβ4-β5 SSB, wherein amino acids 74 to 111 (comprising β4, β45_1_, β45_2_ and β5 strands) were replaced with the corresponding region of *Mtu*SSB, failed to sustain *E. coli* despite its normal oligomerization and DNA binding properties. Another chimera, mβ1-β5 wherein the β1-β5 elements of *Eco*SSB were replaced with the corresponding elements of *Mtu*SSB, conferred filamentation phenotype to *E. coli*. However, the mβ1-β6 SSB with the entire N-terminal domain of *Mtu*SSB (*i. e.* including the β6 strand) fused to the C-terminal domain of *Eco*SSB, functioned well in *E. coli* [22]. These observations suggested specific interaction of β4-β5 region of *Mtu*SSB with the β6 region of *Mtu*SSB. To study the functional importance of such an interaction and to further our understanding of the structure-function relationship of eubacterial SSBs, we generated additional chimeric SSBs (Fig. 2).

The mβ4-β5 SSB was modified to generate mβ4-β5 (acidic), and mβ4-β6 SSBs. One of the distinctive features of the region between the β4 and the β5 strands of *Mtu*SSB is that, unlike *Eco*SSB, it possesses a number of acidic residues (Fig. 1A). Hence, these residues were changed to *Eco*SSB specific sequences in a chimera designated mβ4-β5 (acidic) by mutating E_90_, T_91_, E_95_, K_96_, E_103_, D_105_, and E_106_ within *Mtu*SSB region of β4-β5 to T_90_, D_91_, Q_95_, D_96_, V_103_, N_105_ and V_106_, respectively. To generate mβ4-β6, *Mtu*SSB sequence corresponding to amino acids 74–111 in mβ4-β5 was extended to 131 to include β6 of *Mtu*SSB. Among other constructs, mβ1-α contained the first 73 amino acids (consisting of β1-α structural elements) from *Mtu*SSB and the amino acid 74 to the end from *Eco*SSB. In mβ6 SSB, the β6 strand and the downstream spacer sequences of *Mtu*SSB (amino acid 114 to 133) substituted the corresponding region of *Eco*SSB. The remainder of the sequences (the N-terminal region consisting of the first to 113 amino acids and the C-terminal region (amino acids 134 to the end) were from *Eco*SSB. The mβ6-CTD contains the β6 strand and the C-terminal region (amino acid number 114 to the end) from *Mtu*SSB whereas, the N-terminal region (the first 113 amino acids) from *Eco*SSB. Lastly, the mCTD construct contains only the C-terminal region from *Mtu*SSB (amino acid number 129 to the end) and the N- terminal and the spacer sequences (first 128 amino acids) of *Eco*SSB. More details of generation of these constructs are provided in Methods S1 and Table S1.

### Oligomerization of the chimeric SSBs

**Figure 3:**
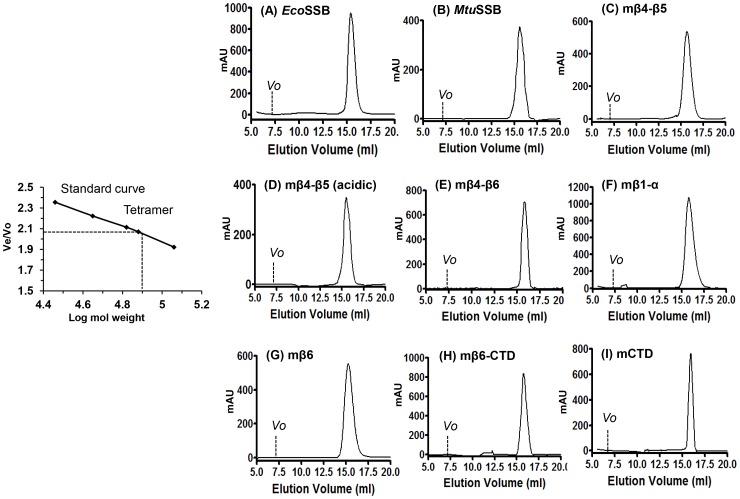
Oligomerization status of SSB proteins. Standard curve *Ve/Vo versus* log molecular size markers is shown in the left most panel. *Ve* represent the peak elution volume of proteins and *Vo* represents the void volume of the column determined using blue dextran (2,000 kDa). Protein size markers [β-galactosidase (116 kDa), elongation factor-G (77 kDa), bovine albumin (66 kDa), egg albumin (44 kDa) and carbonic anhydrase (29 kDa)] were used to make the plot. The tetramer peak corresponding to *Eco*SSB is indicated. Panels (A) to (I) show the gel filtration chromatography elution profiles of SSB proteins. *Vo* and *Ve* of each SSB is indicated.

All SSBs were purified and analyzed by gel filtration chromatography to determine their oligomerization status (Fig. 3). Elution profile of the chimeric SSBs was very similar to those of the wild-type *Eco*SSB and *Mtu*SSB suggesting that they folded properly and formed homotetramers.

### DNA binding properties

**Figure 4:**
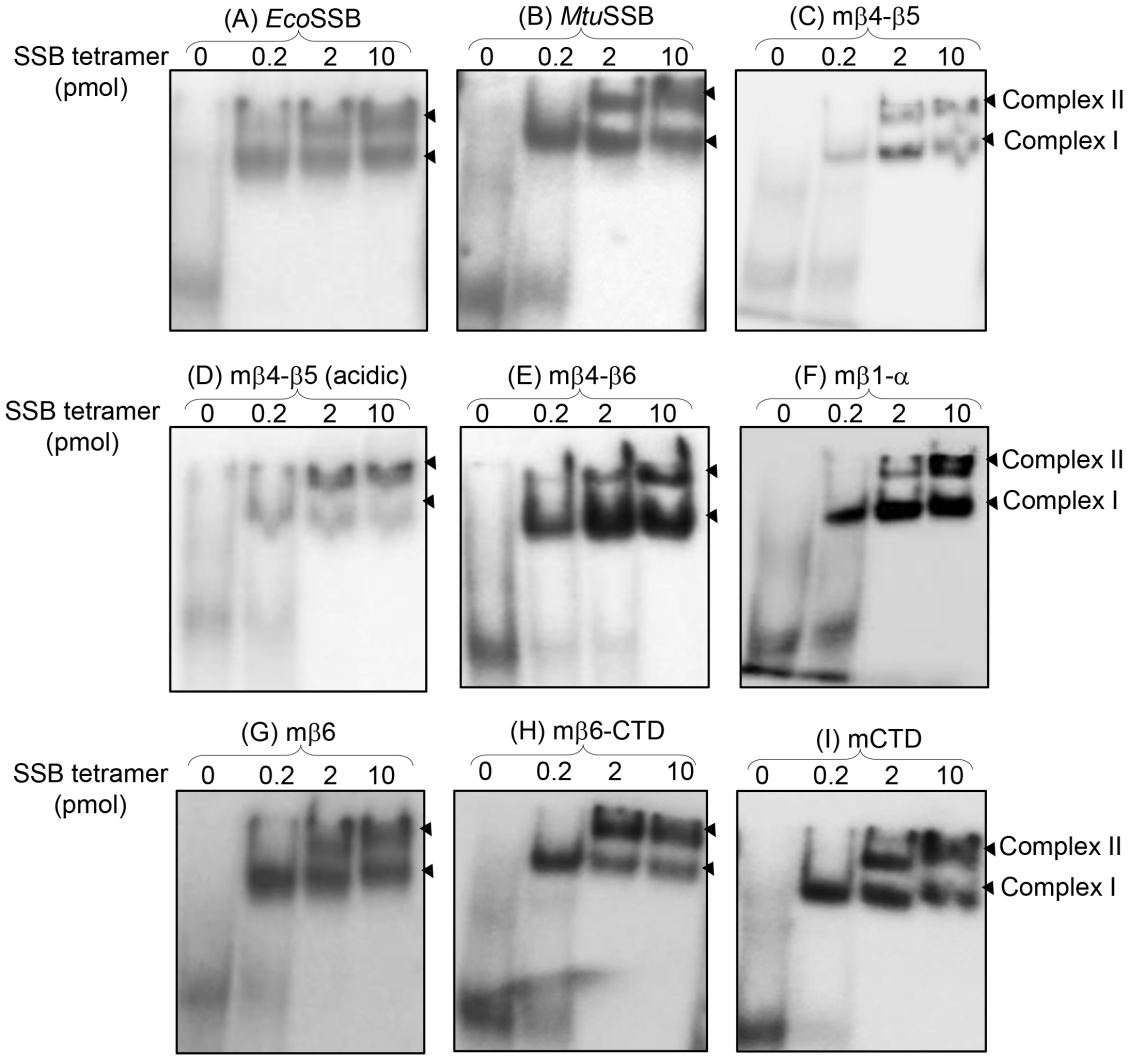
Electrophoretic mobility shift assays using ^32^P labeled 79mer ssDNA. DNA oligomer (1 pmol) was mixed with 0.2 pmol, 2 pmol or 10 pmol SSB tetramer (as indicated) for 30 min and analyzed on native PAGE (8%). DNA binding resulted in ‘Complex I’ at lower protein concentrations and ‘Complex II’ at higher protein concentrations.

To demonstrate the DNA binding abilities of various SSB constructs, we performed electrophoretic mobility shift assays (EMSA) using ^32^P labeled 79mer DNA. Using this assay (Fig. 4), *Eco*SSB and *Mtu*SSB form a faster migrating complex under limiting SSB concentration (Complex I). As the concentration of SSB increases, a second slower migrating band (Complex II) appears. Based on their mobility, these complexes potentially correspond to the SSB_56/65_ and SSB_35_ modes of DNA binding, respectively. More importantly, within the detection limits of this assay, all the chimeric SSBs reveal DNA binding similar to the parent SSBs (compare panels 4A and 4B with 4C to 4H), suggesting that the quaternary structures of the chimeric SSBs are largely unaffected by the mutational manipulations performed to generate them.

### Functionality of SSB chimeras in *E. coli*

Recently, we described a sensitive assay to assess the functionality of a test SSB using a modification of the original ‘plasmid bumping method’ [22], [25]. In the revised assay, the test *ssb* construct (on a ColE1 *ori* plasmid, Amp^R^) is introduced in a *Δssb* (*ssb*::*kan*) strain of *E. coli* (RDP317-1, Kan^R^) harboring a plasmid borne support of wild-type *ssb* on another ColE1 ori plasmid, pHYD*Eco*SSB (Cam^R^). The replication of pHYD*Eco*SSB is dependent on the presence of IPTG. Hence, withdrawal of IPTG from the growth medium results in the loss of the support plasmid (pHYD*Eco*SSB) and failure of the strain growth unless sustained by the test SSB. Growth of the original transformants of the test *ssb* plasmid on plate lacking IPTG, together with the loss of Cam^R^ phenotype, suggests that the test *ssb* complemented the *Δssb* strain of *E. coli* for its function of SSB. An advantage of this assay is that the *in vivo* activity of even a weakly functioning SSB can be assessed (fitness disadvantage of the test *ssb*, if any, is avoided by selectively blocking replication of the original *ssb* support plasmid).

**Figure 5:**
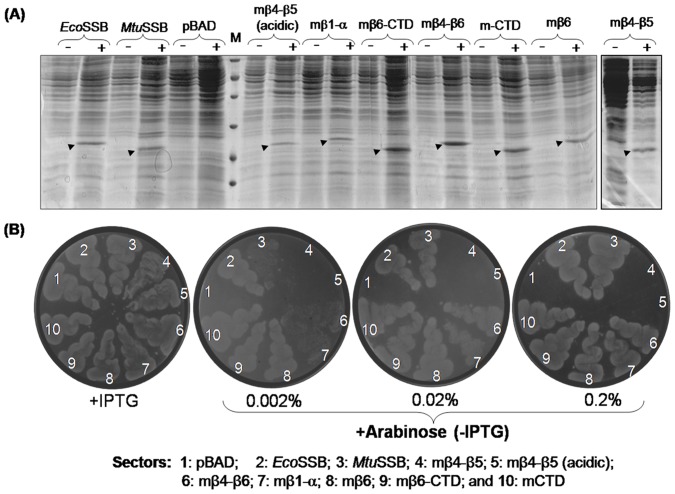
Complementation assays with various SSB constructs. (A) *E. coli* TG1 strains harboring pBAD constructs of SSBs (as shown) were grown to mid log phase in 2–3 ml cultures. Aliquots (1 ml) were either not supplemented (−) or supplemented (+) with 0.02% arabinose, and grown further for 3 h. Cells were harvested and processed as described [22]. Cell-free extracts (∼10 μg total protein) were resolved on SDS-PAGE (15%). (B) Transformants of *E. coli* RDP 317 harboring chimeric SSBs obtained in the presence of IPTG were suspended in LB and streaked on LB-agar (Kan, Amp) containing IPTG or arabinose (0.002–0.2%) and incubated at 37°C for ∼12 h. Sectors: 1, pBAD vector; 2, pBAD*Eco*SSB; 3, pBAD*Mtu*SSB; 4, pBADmβ4-β5; 5, pBADmβ4-β5(acidic); 6, pBADmβ4-β6; 7, pBADmβ1-α; 8, pBADmβ6; 9, pBADmβ6-CTD;10, pBADmCTD.

Using this method, we checked the *in vivo* activity of various SSB constructs subcloned into a ColE1 *ori* (Amp^R^) plasmid wherein their expression was inducible by arabinose (the pBAD series of constructs, Table 1). As shown in Fig. 5A, all constructs showed expression of the corresponding SSBs in *E. coli* TG1. Subsequently, to check for their *in vivo* function, the *ssb* constructs were introduced into RDP317-1 strain (Kan^R^) harboring pHYD*Eco*SSB (Cam^R^), and the transformants were selected on Kan, Amp and 0.02% arabinose plates either containing or lacking IPTG. An analysis of the plating efficiencies (obtained from the ratios of transformants on the –IPTG to +IPTG plates) is shown in Table 2. The mβ4-β5(acidic) SSB did not complement the *Δssb* strain of *E. coli* suggesting that conversion of mβ4-β5 SSB to mβ4-β5(acidic) SSB does not make it functional in *E. coli*. However, transplantation of the β6 region of the *Mtu*SSB into the mβ4-β5 construct in mβ4-β6, resulted in an efficient rescue of the *Δssb* strain of *E. coli* suggesting a functional interaction between the β4-β5 and the β6 regions of *Mtu*SSB. Interestingly, substitution of the unstructured region of *Eco*SSB downstream of its β5 region with the β6 region of *Mtu*SSB in mβ6 SSB, maintained its activity suggesting that the β4-β5 region of *Eco*SSB is tolerant of its downstream sequences.

**Table 2:** *Plating efficiencies of various SSBs.

Strain	Plating efficiency (%)
*Eco*SSB	82±3
*Mtu*SSB	40±4
mβ4-β5	0
mβ4-β5 (acidic)	0
mβ4-β6	49±7
mβ1-α	29±4
mβ6	76±3
mβ6-CTD	48±2
mCTD	46±4

*Plating efficiencies were determined by taking ratios of number of transformants obtained with various SSB constructs in *E. coli* RDP317-1/pHYD*Eco*SSB by plating equal volumes from the same transformation mixes on Kan, Amp and 0.02% arabinose plates *vs* Kan, Amp and IPTG plates. The values have been tabulated from five independent experiments (with three replicates each). Averages with S.D. values are shown.

**Figure 6:**
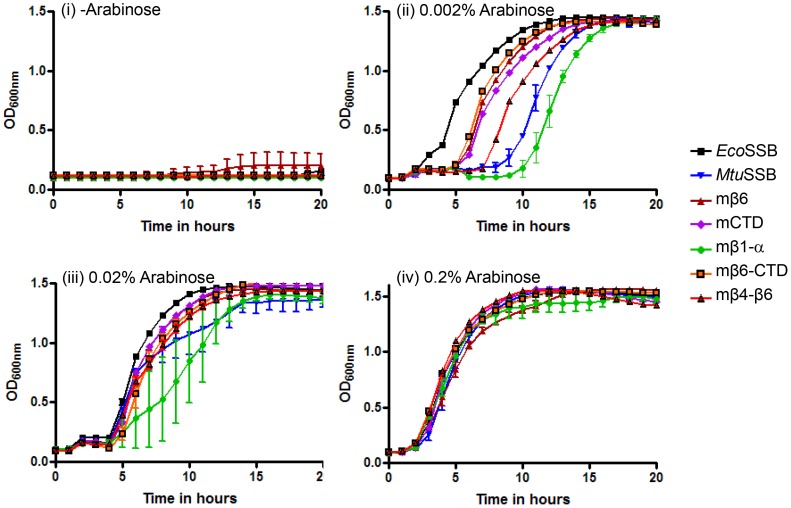
Growth analysis. Growth of *E. coli* RDP317 (Δ*ssb::kan*) supported by various SSBs in the absence (panel i) or presence of 0.002, 0.02% or 0.2% arabinose (panels ii, iii and iv, respectively). Averages of the growth of three independent colonies together with SEM are plotted.

**Figure 7:**
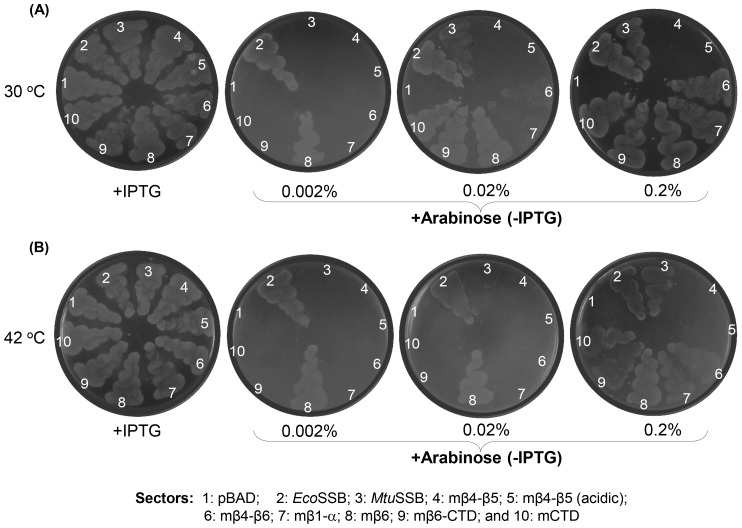
Growth of *E. coli Δssb*::*kan* supported by various SSB constructs at 30°C (A) and 42°C (B). Transformants of *E. coli* RDP 317-1 harboring chimeric SSBs were obtained in the presence of IPTG and processed as in Fig. 5B. Sectors: 1, pBAD vector; 2, pBAD*Eco*SSB; 3, pBAD*Mtu*SSB; 4, pBADmβ4-β5; 5, pBADmβ4-β5(acidic); 6, pBADmβ4-β6; 7, pBADmβ1-α; 8, pBADmβ6; 9, pBADmβ6-CTD;10, pBADmCTD.

*In vivo* complementation by various SSB constructs was further validated by streaking of the freshly obtained transformants (Fig. 5B) on plates containing either IPTG (as control) or varying concentrations of the inducer (0.002–0.2% arabinose). As expected from the replication of the pHYD*Eco*SSB support plasmid in the presence of IPTG, all transformants showed growth on the +IPTG plate. Like the vector control (sector 1), neither the mβ4-β5 nor the mβ4-β5 (acidic) complemented the *Δssb* strain at any of the arabinose concentrations (sectors 4 and 5). Further, the results of the growth curve analyses (Fig. 6) of the strains harboring SSBs that sustained *E. coli* are also consistent with the plating efficiency data. Weakly functioning SSBs, in general, resulted in longer lag phases when expression of SSBs was induced with 0.002% arabinose (panel ii). These differences were, however, lost in cultures induced with 0.02% or 0.2% arabinose (panels iii and iv) which result in higher level of expression of these SSBs (Fig. S1). As a control, when the growth curve analyses were carried out in the absence of inducer, arabinose (Fig. 6, panel i) none of the cultures grew confirming that the phenotypes observed in Table 2, and Figs. 5B and 6 (panels ii and iii) are due to the plasmid borne SSBs. The longer lag phases in Fig. 6 (panel ii) could be a stress related phenomenon. Interestingly, we observed that the weakly functioning SSBs also conferred temperature and cold sensitive phenotypes to *E. coli* for growth at 42°C and 30°C, respectively (Fig. 7). These phenotypes could also be suppressed upon induction of SSB expression with higher concentrations of arabinose. It may also be noted that even under these conditions (temperatures of 42°C or 30°C), the mβ4-β5 and mβ4-β5 (acidic) failed to complement the *E. coliΔssb* strain (Figs. 7A and 7B, sectors 4 and 5, respectively).

### Microscopic analyses

**Figure 8:**
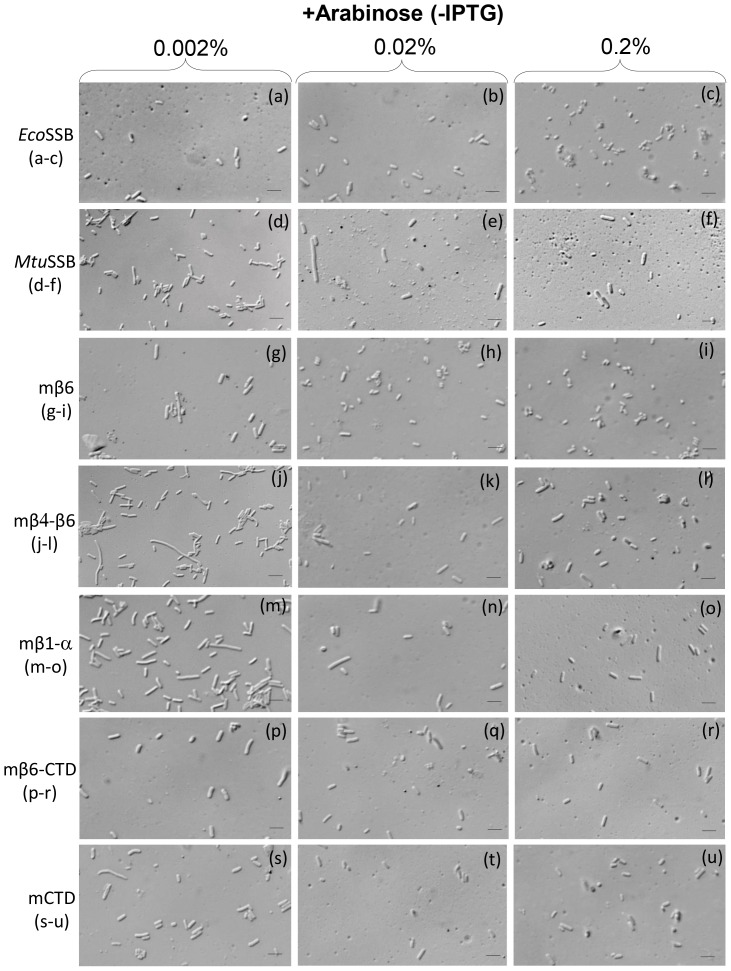
Microscopic observations of *E. coli Δssb*::*kan* supported by various SSB constructs. Cultures of *E. coli* RDP317 (Δ*ssb::kan*) transformants harboring various SSB constructs were grown in the presence of indicated concentrations of arabinose and analyzed by phase contrast microscopy. Bars at the lower left of each panel indicate a scale of 2 μm.

In our earlier study microscopic analyses of the fixed *E. coli* cells revealed that the mβ1-β5 SSB, a poorly functioning SSB, resulted in a notable filamentation phenotype [22]. On the other hand, SSBs that functioned, but not as well as *Eco*SSB, resulted in a slightly elongated cell phenotype. As before, *Mtu*SSB showed a phenotype of slightly elongated cells (Fig. 8, compare panels d and a). However, the mβ4-β6 SSB showed a more pronounced phenotype of the elongated cells (compare panel j with a). The mβ1-α SSB showed a weak phenotype of the elongated cells (compare panels m with a). Interestingly, as in Figs. 6 and 7, overexpression of the SSBs suppressed these phenotypes (compare panels d with e and f; j with k and l; m with n and o).

## Discussion

Determination of the three-dimensional structure of *Mtu*SSB by X-ray crystallography revealed that while its structure at the tertiary level is very similar to that of *Eco*SSB, it shows significant variations at the level of quaternary interactions [19]. A notable difference seen at the level of tetramerization of *Mtu*SSB is the presence of a clamp like structure formed by the β6 strand of the mycobacterial SSB [19]. However, it has so far remained unclear as to what the biological significance of this unique structural element of *Mtu*SSB is.

The L_45_ loop in *Eco*SSB has been shown to undergo a conformational change upon DNA binding and suggested to be important for its cooperative binding [17], [18]. In addition, the computational analyses suggested that the movements of L_45_ loop in *Eco*SSB, *Mtu*SSB, and *Streptomyces coelicolor* SSB are different [21]. Our observation shows that the mβ4-β5 construct wherein the L_45_ loop (of *Mtu*SSB origin) is intact does not function in *E. coli* but the mβ4-β6 SSB wherein a small region (β6) downstream of β5 was also included, does. Together with the biophysical and computational analyses [17], [18], [19], [21], these observations highlight the importance of the functional interactions of the L_45_ loop with the β6 region. And, some of these interactions may well contribute to the stability of the *Mtu*SSB tetramer predicted from the crystal structure analysis [19]. However, it should also be said that our present study does not allow us to comment on the mechanistic details of such interactions for the SSB function *in vivo*.

How crucial is the species specificity of these interactions (in the context of SSB tetramer) for SSB function? When we changed this region of *Eco*SSB with the corresponding region of *Mtu*SSB in the context of *E. coli* L_45_ loop, we did not detect a significant defect in the chimeric SSB (mβ6), suggesting that the interactions of the L_45_ loop with its downstream sequence are more tolerant in *Eco*SSB. In the context of *M. tuberculosis* L_45_, when the entire upstream region of *Mtu*SSB was provided, such as in the mβ1-β5 SSB *i. e.*, wherein the N-terminal domain (β1-β5) of *Eco*SSB was replaced with the corresponding sequence from *Mtu*SSB, it did sustain *E. coli* viability but the growth was poor and it resulted in a filamentation phenotype [22]. These observations suggest that the context of both the upstream and the downstream regions (with respect to the L_45_ loop of *Mtu*SSB) is biologically significant. Lack of either of the regions compromises SSB function in a context dependent manner. However, the chimeras mβ1-α and mβ6-CTD, wherein the entire region upstream of, or downstream of the loop L_45_ (of *Eco*SSB), respectively are from *Mtu*SSB, functioned well in *E. coli* (as did the mβ6). These observations suggest that in *Eco*SSB, any interactions mediated by the L_45_ are more tolerant of the neighboring sequences. This is further indicated by the observation (Fig. 6, panel ii) that the construct mCTD (*Eco*SSB harboring only the CTD from *Mtu*SSB) functioned nearly as well as the mβ6 (harboring only the β6 of *Mtu*SSB) or the mβ6-CTD (harboring the entire region downstream of L_45_, from *Mtu*SSB). An availability of the three-dimensional structures of the chimeric SSBs may further our understanding of the interactions L_45_ establishes within SSB.

Finally, the modification [22] of the ‘plasmid bumping’ assay [25] we recently developed has been useful in determining the efficacy of SSB mutants in sustaining *E. coli* even when they are compromised in their function, and provided with a convenient approach to study the structure-function relationship of the various structural elements of the eubacterial SSBs.

## Supporting Information

Figure S1 SSB expression in response to increasing arabinose concentration in the medium.
(DOC)

Table S1 Nucleotide and amino acid sequences of the *Eco*SSB, *Mtu*SSB and various chimeric SSBs.
(DOC)

Methods S1 Details of chimeric SSB constructions.
(DOC)
