# Supplementary material for: A Genetic Analysis of the Functional Interactions within Mycobacterium tuberculosis Single-Stranded DNA Binding Protein
Source: PLoS One. 2014 Apr 10;9(4):e94669. doi: 10.1371/journal.pone.0094669 (PMC3983218; doi:10.1371/journal.pone.0094669)
Supplement: Table S1 — Nucleotide and amino acid sequences of the EcoSSB, MtuSSB and various chimeric SSBs. (DOC) [file pone.0094669.s002.doc]

**Table S1: Nucleotide and amino acid sequences of the SSB constructs**

| ***Eco*SSB** |
| --- |
| **atggccagcagaggcgtaaacaaggttattctcgttggtaatctgggtcaggacccggaa**  **M A S R G V N K V I L V G N L G Q D P E 20**  **gtacgctacatgccaaatggtggcgcagttgccaacattacgctggctacttccgaatcc**  **V R Y M P N G G A V A N I T L A T S E S 40**  **tggcgtgataaagcgaccggcgagatgaaagaacagactgaatggcaccgcgttgtgctg**  **W R D K A T G E M K E Q T E W H R V V L 60**  **ttcggcaaactggcagaagtggcgagcgaatatctgcgtaaaggttctcaggtttatatc**  **F G K L A E V A S E Y L R K G S Q V Y I 80**  **gaaggtcagctgcgtacccgtaaatggaccgatcaatccggtcaggatcgctacaccaca**  **E G Q L R T R K W T D Q S G Q D R Y T T 100**  **gaagtcgtggtgaacgttggcggcaccatgcagatgctgggtggtcgtcagggtggtggc**  **E V V V N V G G T M Q M L G G R Q G G G 120**  **gctccggcaggtggcaatatcggtggtggtcagccgcagggcggttggggtcagcctcag**  **A P A G G N I G G G Q P Q G G W G Q P Q 140**  **cagccgcagggtggcaatcagttcagcggcggcgcgcagtctcgcccgcagcagtccgct**  **Q P Q G G N Q F S G G A Q S R P Q Q S A 160**  **ccggcagcgccgtctaacgagccgccgatggactttgatgatgacattccgttctga**  **P A A P S N E P P M D F D D D I P F - 178** |
| ***Mtu*SSB** |
| **atggctggtgacaccaccatcaccatcgtcggaaatctgaccgctgaccccgagctgcgg**  **M A G D T T I T I V G N L T A D P E L R 20**  **ttcaccccgtccggtgcggccgtggcgaatttcaccgtggcgtcaacgccccggatctat**  **F T P S G A A V A N F T V A S T P R I Y 40**  **gaccgtcagaccggcgaatggaaagacggcgaagcgctgttcctccggtgcaatatctgg**  **D R Q T G E W K D G E A L F L R C N I W 60**  **cgggaggcggccgagaacgtggccgagagcctcacccggggggcacgagtcatcgttagc**  **R E A A E N V A E S L T R G A R V I V S 80**  **gggcggcttaagcagcggtcgtttgaaacccgtgagggcgagaagcgcaccgtcatcgag**  **G R L K Q R S F E T R E G E K R T V I E 100**  **gtcgaggtcgatgagattgggccttcgcttcggtacgccaccgccaaggtcaacaaggcc**  **V E V D E I G P S L R Y A T A K V N K A 120**  **agccgcagcggcgggtttggcagcggatcccgtccggcgccggcgcagaccagcagcgcc**  **S R S G G F G S G S R P A P A Q T S S A 140**  **tcgggagatgacccgtggggcagcgcaccggcgtcgggttcgttcggcggcggcgatgac**  **S G D D P W G S A P A S G S F G G G D D 160**  **gaaccgccattctga**  **E P P F - 164** |
| **mβ4-β5** |
| **atggccagcagaggcgtaaacaaggttattctcgttggtaatctgggtcaggacccggaa**  **M A S R G V N K V I L V G N L G Q D P E 20**  **gtacgctacatgccaaatggtggcgcagttgccaacattacgctggctacttccgaatcc**  **V R Y M P N G G A V A N I T L A T S E S 40**  **tggcgtgataaagcgaccggcgagatgaaagaacagactgaatggcaccgcgttgtgctg**  **W R D K A T G E M K E Q T E W H R V V L 60**  **ttcggcaaactggcagaagtggcgagcgaatatctggcccggggggcacgagtcatcgtt**  **F G K L A E V A S E Y L A R G A R V I V 80**  **agcgggcggcttaagcagcggtcgtttgaaacccgtgagggcgagaagcgcaccgtcatc**  **S G R L K Q R S F E T R E G E K R T V I 100**  **gaggtcgaggtcgatgagattgggccttcgctagctggtcgtcagggtggtggcgctccg**  **E V E V D E I G P S L A G R Q G G G A P 120**  **gcaggtggcaatatcggtggtggtcagccgcagggcggttggggtcagcctcagcagccg**  **A G G N I G G G Q P Q G G W G Q P Q Q P 140**  **cagggtggcaatcagttcagcggcggcgcgcagtctcgcccgcagcagtccgctccggca**  **Q G G N Q F S G G A Q S R P Q Q S A P A 160**  **gcgccgtctaacgagccgccgatggactttgatgatgacattccgttctga**  **A P S N E P P M D F D D D I P F - 176** |
| **mβ4-β5 (acidic)** |
| **atggccagcagaggcgtaaacaaggttattctcgttggtaatctgggtcaggacccggaa**  **M A S R G V N K V I L V G N L G Q D P E 20**  **gtacgctacatgccaaatggtggcgcagttgccaacattacgctggctacttccgaatcc**  **V R Y M P N G G A V A N I T L A T S E S 40**  **tggcgtgataaagcgaccggcgagatgaaagaacagactgaatggcaccgcgttgtgctg**  **W R D K A T G E M K E Q T E W H R V V L 60**  **ttcggcaaactggcagaagtggcgagcgaatatctggcccggggggcacgagtcatcgtt**  **F G K L A E V A S E Y L A R G A R V I V 80**  **agcgggcggcttaagcagcggtcgtttacagaccgttcgggccaggaccgcaccgtcatc**  **S G R L K Q R S F T D R S G Q D R T V I 100**  **gaggtcgtggtcaatgtgattgggccttcgctagctggtcgtcagggtggtggcgctccg**  **E V V V N V I G P S L A G R Q G G G A P 120**  **gcaggtggcaatatcggtggtggtcagccgcagggcggttggggtcagcctcagcagccg**  **A G G N I G G G Q P Q G G W G Q P Q Q P 140**  **cagggtggcaatcagttcagcggcggcgcgcagtctcgcccgcagcagtccgctccggca**  **Q G G N Q F S G G A Q S R P Q Q S A P A 160**  **gcgccgtctaacgagccgccgatggactttgatgatgacattccgttctga**  **A P S N E P P M D F D D D I P F - 176** |
| | **mβ4-β6** | | --- | | **atggccagcagaggcgtaaacaaggttattctcgttggtaatctgggtcaggacccggaa**  **M A S R G V N K V I L V G N L G Q D P E 20**  **gtacgctacatgccaaatggtggcgcagttgccaacattacgctggctacttccgaatcc**  **V R Y M P N G G A V A N I T L A T S E S 40**  **tggcgtgataaagcgaccggcgagatgaaagaacagactgaatggcaccgcgttgtgctg**  **W R D K A T G E M K E Q T E W H R V V L 60**  **ttcggcaaactggcagaagtggcgagcgaatatctggcccggggggcacgagtcatcgtt**  **F G K L A E V A S E Y L A R G A R V I V 80**  **agcgggcggcttaagcagcggtcgtttgaaacccgtgagggcgagaagcgcaccgtcatc**  **S G R L K Q R S F E T R E G E K R T V I 100**  **gaggtcgaggtcgatgagattgggccttcgctagcgtacgccaccgccaaggtcaacaag**  **E V E V D E I G P S L A Y A T A K V N K 120**  **gccagccgcagcggcgggtttggcagcggatcccagccgcagggcggttggggtcagcct**  **A S R S G G F G S G S Q P Q G G W G Q P 140**  **cagcagccgcagggtggcaatcagttcagcggcggcgcgcagtctcgcccgcagcagtcc**  **Q Q P Q G G N Q F S G G A Q S R P Q Q S 160**  **gctccggcagcgccgtctaacgagccgccgatggactttgatgatgacattccgttctga**  **A P A A P S N E P P M D F D D D I P F - 179** |   **mβ1-α** |
| **atggctggtgacaccaccatcaccatcgtcggaaatctgaccgctgaccccgagctgcgg**  **M A G D T T I T I V G N L T A D P E L R 20**  **ttcaccccgtccggtgcggccgtggcgaatttcaccgtggcgtcaacgccccggatctat**  **F T P S G A A V A N F T V A S T P R I Y 40**  **gaccgtcagaccggcgaatggaaagacggcgaagcgctgttcctccggtgcaatatctgg**  **D R Q T G E W K D G E A L F L R C N I W 60**  **cgggaggcggccgagaacgtggccgagagcctcacccggggttctcaggtttatatcgaa**  **R E A A E N V A E S L T R G S Q V Y I E 80**  **ggtcagctgcgtacccgtaaatggaccgatcaatccggtcaggatcgctacaccacagaa**  **G Q L R T R K W T D Q S G Q D R Y T T E 100**  **gtcgtggtgaacgttggcggcaccatgcagatgctgggtggtcgtcagggtggtggcgct**  **V V V N V G G T M Q M L G G R Q G G G A 120**  **ccggcaggtggcaatatcggtggatcccagccgcagggcggttggggtcagcctcagcag**  **P A G G N I G G S Q P Q G G W G Q P Q Q 140**  **ccgcagggtggcaatcagttcagcggcggcgcgcagtctcgcccgcagcagtccgctccg**  **P Q G G N Q F S G G A Q S R P Q Q S A P 160**  **gcagcgccgtctaacgagccgccgatggactttgatgatgacattccgttctga**  **A A P S N E P P M D F D D D I P F - 177** |
| **mβ6** |
| **atggccagcagaggcgtaaacaaggttattctcgttggtaatctgggtcaggacccggaa**  **M A S R G V N K V I L V G N L G Q D P E 20**  **gtacgctacatgccaaatggtggcgcagttgccaacattacgctggctacttccgaatcc**  **V R Y M P N G G A V A N I T L A T S E S 40**  **tggcgtgataaagcgaccggcgagatgaaagaacagactgaatggcaccgcgttgtgctg**  **W R D K A T G E M K E Q T E W H R V V L 60**  **ttcggcaaactggcagaagtggcgagcgaatatctgcgtaaaggttctcaggtttatatc**  **F G K L A E V A S E Y L R K G S Q V Y I 80**  **gaaggtcagctgcgtacccgtaaatggaccgatcaatccggtcaggatcgctacaccaca**  **E G Q L R T R K W T D Q S G Q D R Y T T 100**  **gaagtcgtggtgaacgttggcggcaccatgcagatgctagcgtacgccaccgccaaggtc**  **E V V V N V G G T M Q M L A Y A T A K V 120**  **aacaaggccagccgcagcggcgggtttggcagcggatcccagccgcagggcggttggggt**  **N K A S R S G G F G S G S Q P Q G G W G 140**  **cagcctcagcagccgcagggtggcaatcagttcagcggcggcgcgcagtctcgcccgcag**  **Q P Q Q P Q G G N Q F S G G A Q S R P Q 160**  **cagtccgctccggcagcgccgtctaacgagccgccgatggactttgatgatgacattccg**  **Q S A P A A P S N E P P M D F D D D I P 180**  **ttctga 181**  **F -** |
| **mβ6-CTD** |
| **atggccagcagaggcgtaaacaaggttattctcgttggtaatctgggtcaggacccggaa**  **M A S R G V N K V I L V G N L G Q D P E 20**  **gtacgctacatgccaaatggtggcgcagttgccaacattacgctggctacttccgaatcc**  **V R Y M P N G G A V A N I T L A T S E S 40**  **tggcgtgataaagcgaccggcgagatgaaagaacagactgaatggcaccgcgttgtgctg**  **W R D K A T G E M K E Q T E W H R V V L 60**  **ttcggcaaactggcagaagtggcgagcgaatatctgcgtaaaggttctcaggtttatatc**  **F G K L A E V A S E Y L R K G S Q V Y I 80**  **gaaggtcagctgcgtacccgtaaatggaccgatcaatccggtcaggatcgctacaccaca**  **E G Q L R T R K W T D Q S G Q D R Y T T 100**  **gaagtcgtggtgaacgttggcggcaccatgcagatgctagcgtacgccaccgccaaggtc**  **E V V V N V G G T M Q M L A Y A T A K V 120**  **aacaaggccagccgcagcggcgggtttggcagcggatcccgtccggcgccggcgcagacc**  **N K A S R S G G F G S G S R P A P A Q T 140**  **agcagcgcctcgggagatgacccgtggggcagcgcaccggcgtcgggttcgttcggcggc**  **S S A S G D D P W G S A P A S G S F G G 160**  **ggcgatgacgaaccgccattctga**  **G D D E P P F - 167** |
| **mCTD** |
| **atggccagcagaggcgtaaacaaggttattctcgttggtaatctgggtcaggacccggaa**  **M A S R G V N K V I L V G N L G Q D P E 20**  **gtacgctacatgccaaatggtggcgcagttgccaacattacgctggctacttccgaatcc**  **V R Y M P N G G A V A N I T L A T S E S 40**  **tggcgtgataaagcgaccggcgagatgaaagaacagactgaatggcaccgcgttgtgctg**  **W R D K A T G E M K E Q T E W H R V V L 60**  **ttcggcaaactggcagaagtggcgagcgaatatctgcgtaaaggttctcaggtttatatc**  **F G K L A E V A S E Y L R K G S Q V Y I 80**  **gaaggtcagctgcgtacccgtaaatggaccgatcaatccggtcaggatcgctacaccaca**  **E G Q L R T R K W T D Q S G Q D R Y T T 100**  **gaagtcgtggtgaacgttggcggcaccatgcagatgctgggtggtcgtcagggtggtggc**  **E V V V N V G G T M Q M L G G R Q G G G 120**  **gctccggcaggtggcaatatcggtggatcccgtccggcgccggcgcagaccagcagcgcc**  **A P A G G N I G G S R P A P A Q T S S A 140**  **tcgggagatgacccgtggggcagcgcaccggcgtcgggttcgttcggcggcggcgatgac**  **S G D D P W G S A P A S G S F G G G D D 160**  **gaaccgccattctga**  **E P P F - 164** |

**Note:** Sequences shown in green and red are of *Eco*SSB and *Mtu*SSB origin, respectively. Sequences shown in blue are mutations incorporated to create a desired restriction site to generate the chimeric construct.
