# Supplementary material for: A Genetic Analysis of the Functional Interactions within Mycobacterium tuberculosis Single-Stranded DNA Binding Protein
Source: PLoS One. 2014 Apr 10;9(4):e94669. doi: 10.1371/journal.pone.0094669 (PMC3983218; doi:10.1371/journal.pone.0094669)
Supplement: Methods S1 — Details of chimeric SSB constructions. (DOC) [file pone.0094669.s003.doc]

**Methods S1**

**Generation of SSB constructs**

**(a) pBADmβ4-β5 (acidic):** The amino acids _87_RSFETREGEKRTVIEVEVDEIG_108_ in the β4-β5 region of mβ4-β5 SSB^22^ were changed to _87_RSFTDRSGQDRTVIEVVVNVIG_108_ using mβ4-β5 acidic-Fp and mβ4-β5 acidic-Rp primers. The condition for inverse PCR (50 μl) included heating at 94 ^o^C; followed by 24 cycles of denaturation at 94 ^o^C for 1 min, annealing at 48 ^o^C for 45 s, and polymerization at 70 ^o^C for 10 min and a final extension at 70 ^o^C for 10 min. The reaction was subjected to overnight treatment with 10 units of DpnI. A 10 μl aliquot was used to transform *E. coli* TG1. The desired clones of mβ4-β5(acidic) SSB were identified and confirmed by DNA sequencing of the plasmid minipreparations.

**(b) pBADmβ6-CTD:** This construct consists of initial 113 amino acids from *Eco*SSB (M_1_-L_113_) and 114 to 167 amino acids from *Mtu*SSB (R111A to F_164_ of *Mtu*SSB). pTrc*Mtu*SSB-NheI was digested with NheI and HindIII, and the released fragment was ligated with the vector backbone released upon NheI and HindIII digestion of pTrc*Eco*SSB-NheI [22] to generate pTrc-mβ6-CTD. The pTrc-mβ6-CTD was digested with NcoI and HindIII, and the released fragment was subcloned into similarly digested pBAD/HisB to generate pBAD mβ6-CTD and confirmed by DNA sequencing.

**(c) pBADmβ6 SSB:** This construct contains initial 113 amino acid from *Eco*SSB, 114 to 133 from *Mtu*SSB (A_111_ to S_130_ of *Mtu*SSB). BamHI and HindIII digested fragment of pBAD *Eco*SSB containing NheI and BamHI^22^ was ligated with vector backbone of BamHI and HindIII digested pBAD mβ6-CTD. The clones were confirmed by DNA sequencing.

**(d) pBADmCTD:** This constructs consists initial 128 amino acids from *Eco*SSB (M_1_-G_128_) and 129 to 164 amino acids from *Mtu*SSB (G_129_ to F_164_). *Mtu*SSB contains unique BamHI site (corresponding to amino acid position 129 of *Eco*SSB). A BamHI site was generated in *Eco*SSB by mutating G129S^26^. C-terminal region of *Eco*SSB was swapped with C-terminal of *Mtu*SSB to generate mCTD SSB.

**(e) pBADmβ4-β6:** NheI and PvuI digested pBADmβ6 SSB (insert) was ligated to similarly digested pBADmβ4-β5 SSB (vector) [22]. The clones obtained were confirmed by DNA sequencing.

**(f) pBADmβ1-α:** In this chimeric SSB, β1-α regions of *Eco*SSB (M_1_- G_75_) was substituted with the corresponding regions from *Mtu*SSB (M_1_-G_74_). The DNA sequence, ACCCGGGGG encoding _72_TRG_74_ and possessing an XmaI site (CCCGGG) is present in *Mtu*SSB. The *Eco*SSB-XmaI Fp was used to mutate the CGTAAAGGT sequence of *Eco*SSB encoding _73_RKG_75_ to an XmaI site (GCCCGGGGT) encoding _73_ARG_75_. *Eco*SSB-XmaI-Fp and pTrcBcl-Rp were used to amplify ~330 bp of *Eco*SSB consisting of (amino acids _68_S-F_177_). The PCR conditions were, initial denaturation at 94 ^o^C for 4 min, followed by 24 cycles of denaturation at 94 ^o^C for 1 min, annealing at 50 ^o^C for 30 s, polymerization at 70 ^o^C for 45 s. PCR was finally extended at 70 ^o^C for 10 min. The PCR product was digested with XmaI and HindIII and ligated into similarly digested pBAD *Mtu*SSB. The clones were confirmed by DNA sequencing.
