# Supplementary material for: A Genetic Analysis of the Functional Interactions within Mycobacterium tuberculosis Single-Stranded DNA Binding Protein
Source: PLoS One. 2014 Apr 10;9(4):e94669. doi: 10.1371/journal.pone.0094669 (PMC3983218; doi:10.1371/journal.pone.0094669)
Supplement: Supplementary file 3 [file pone.0094669.s001.doc]

Rex et. al., Fig. S1

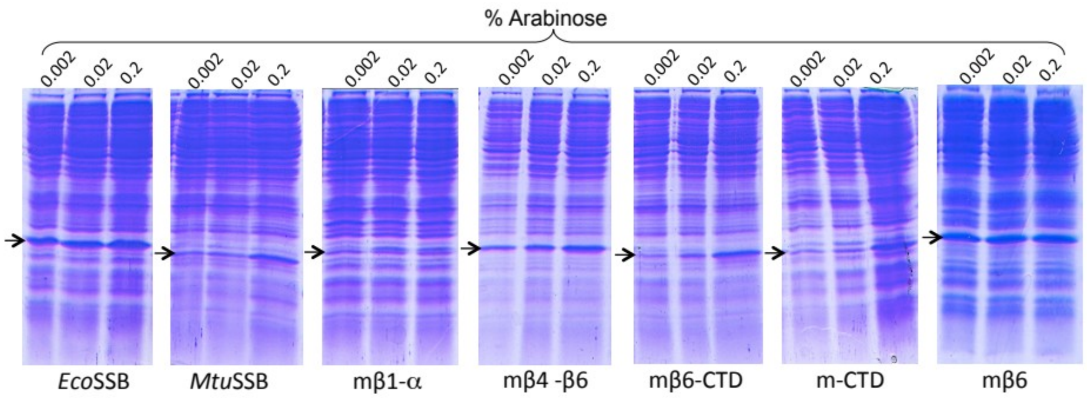

Figure S1: *E. coli* RDP317-1 (Δ*ssb::kan*) harboring pBAD constructs of of the indicated SSBs were grown in increasing concentrations of arabinose (0.002, 0.02, 0.2%, as indicated) in 2-3 ml cultures until saturation. Cells were harvested by centrifugation, resuspended in 250 µl TME (Tris-HCl, pH 8.0, 2 mM β-mercaptoethanol and 1 mM Na_2EDTA), subjected to sonication (20 s pulses on/off thrice) and spun at 13000 rpm for 20 minutes at 4 °C. The cell-free extracts (~15 µg total protein) were resolved on 15% SDS PAGE. SSB bands are indicated by arrows.
